# Scutellarin inhibits ferroptosis by promoting cellular antioxidant capacity through regulating Nrf2 signaling: Scutellarin inhibits ferroptosis by promoting cellular antioxidant capacity

**DOI:** 10.3724/abbs.2025112

**Published:** 2025-08-19

**Authors:** Haiyan Yang, Onkei Chan, Xiaodi Huang, Liang Yan, Nuo Sun, Yaping Li, Zijian Shi, Qingbing Zha, Dongyun Ouyang, Jinhua Li, Xianhui He

**Affiliations:** 1 Department of Immunology and Microbiology College of Life Science and Technology Jinan University Guangzhou 510632 China; 2 Guangdong Provincial Key Laboratory of Spine and Spinal Cord Reconstruction the Fifth Affiliated Hospital of Jinan University (Heyuan Shenhe People’s Hospital) Heyuan 517000 China; 3 Center of Reproductive Medicine the First Affiliated Hospital of Jinan University Guangzhou 510632 China; 4 Department of Fetal Medicine the First Affiliated Hospital of Jinan University Guangzhou 510632 China; 5 Department of Ultrasound the First Affiliated Hospital of Jinan University Guangzhou 510632 China; 6 Department of Ultrasound the Fifth Affiliated Hospital of Jinan University (Heyuan Shenhe People’s Hospital) Heyuan 517000 China

**Keywords:** acute kidney injury, ferroptosis, mitochondrial dysfunction, Nrf2, scutellarin

## Abstract

Ferroptosis is a lytic form of regulated cell death that is driven by iron-dependent lipid peroxidation and has been implicated in various diseases, including acute kidney injury (AKI). Scutellarin is a flavonoid isolated from *Erigeron breviscapus* (Vant.) Hand.-Mazz. and possesses various pharmacological activities, including anti-inflammatory and antioxidative properties. However, it is unclear whether scutellarin can inhibit ferroptosis and mitigate related diseases. In this study, we show that scutellarin can inhibit ferroptosis in both human HK-2 cells and mouse bone marrow-derived macrophages stimulated with RSL3 or erastin. Mitochondrial dysfunction and reactive oxygen species generation are counteracted by scutellarin treatment, suggesting the involvement of its antioxidative activity. Furthermore, scutellarin increases the nuclear levels of Nrf2 and the expressions of its target genes, including *HO-1* and *GPX4*. Scutellarin-mediated inhibition of ferroptosis and increases in these proteins are abrogated by co-treatment with brusatol, an Nrf2 inhibitor, indicating an essential role for Nrf2 in this process. In a mouse model of folic acid-induced AKI, scutellarin mitigates acute renal damage, as revealed by histopathological analysis and serum blood urea nitrogen and creatinine assays. Folic acid-induced acute renal injury is associated with increased ferroptosis, as revealed by elevated level of 4-hydroxynonenal (4-HNE), a surrogate marker of ferroptosis, which is diminished by scutellarin co-treatment. Specifically, the elevated 4-HNE levels in macrophages (MAC-2 positive) and other renal cells are suppressed by scutellarin. Overall, scutellarin can inhibit ferroptosis both in cultured cells and in a mouse model of AKI by regulating Nrf2 signaling.

## Introduction

Ferroptosis is a nonapoptotic form of regulated cell death that is driven by iron-dependent lipid peroxidation [ 1– 4] . The (phosphor)lipid peroxide-reducing enzyme glutathione peroxidase 4 (GPX4) is a central regulator of ferroptosis and prevents cell death by catalyzing the conversion of lipid peroxides into non-toxic lipid alcohols in the presence of sufficient glutathione [ 2, 5, 6] . The cystine/glutamate antiporter (X _c_^–^ also known as xCT) system is essential for maintaining the level of intracellular glutathione [6]. In addition to the system X _c_^–^/GPX4 cellular antioxidant axis, ferroptosis suppressor protein 1 (FSP1) is the second mainstay of ferroptosis-inhibiting antioxidant enzyme. Mechanistically, FSP1 is recruited to the plasma membrane by myristoylation and acts as an oxidoreductase to reduce ubiquinone (also known as coenzyme Q _10_) into its reduced form ubiquinol, which functions as a lipophilic radical trapping antioxidant to block lipid peroxides [ 7, 8] . In addition to mitochondrial GPX4, dihydroorotate dehydrogenase (DHODH) has been found to attenuate ferroptosis in mitochondria by reducing ubiquinone to ubiquinol [9]. Ferroptosis is also regulated by small antioxidative molecules such as α-tocopherol (vitamin E), which act as lipophilic radical-trapping antioxidants to terminate the propagation of lipid peroxides [ 3, 5] . Moreover, two recent reports showed that 7-hydrocholesterol is an endogenous suppressor of ferroptosis that acts as an antioxidant to protect against lipid peroxidation, thereby regulating the sensitivity of cells to ferroptosis [ 10, 11] . Notably, several important antioxidant proteins, such as GPX4 and heme oxygenase-1 (HO-1), are encoded by target genes of nuclear factor erythroid 2-related factor 2 (Nrf2), indicating a critical role of Nrf2 in modulating ferroptosis [ 12, 13] . Thus, the antioxidant systems of a cell play critical roles in protecting against ferroptosis.

Ferroptosis, an oxidative form of cell death, can be induced by the disruption of cellular antioxidant systems through the inhibition of GPX4 activity by genetic deletion of this gene or by RSL3-induced GPX4 inactivation [5]. This form of cell death can also be triggered by depletion of glutathione through inhibition of system X _c_^-^, ^,^ thereby indirectly decreasing GPX4 activity [ 1, 5] . Although many studies have adopted such biochemical methods to induce ferroptosis *in vitro* in cultured cells, emerging evidence implicates this oxidative form of regulated cell death in various pathological conditions, including degenerative diseases, cancers, and organ injury [ 4, 6, 14] . Deletion of GPX4 results in acute renal failure in mice, indicating an essential role for the glutathione/GPX4 axis in suppressing lipid peroxidation-induced acute kidney injury (AKI) and associated death [ 15, 16] . Several studies in mouse models of AKI have revealed the prominent role of ferroptosis in the pathology of renal tubular injury, indicating that targeting ferroptosis can improve the survival of mice with AKI [ 16, 17] . Interestingly, 7-hydrocholesterol administration protects the kidney from ischemia-reperfusion injury through the inhibition of ferroptosis [10]. Therefore, targeting ferroptosis is a promising avenue for the treatment of renal injury during AKI.

Scutellarin is a flavonoid isolated from *Erigeron breviscapus* (Vant.) Hand.-Mazz. [18], a traditional medicinal herb that has long been used to treat paralysis after stroke and joint pain in rheumatoid arthritis by Yi minority people in southwestern China [19]. Scutellarin is the major active ingredient of this herbal medicine [18]. Breviscapine, which contains ≥ 90% scutellarin, has been used clinically for the treatment of cardiovascular and cerebrovascular diseases [20]. Other clinical studies have shown that breviscapine can improve hypertension, hyperlipidemia, diabetic peripheral neuropathy, and diabetic nephropathy [19]. The therapeutic properties of breviscapine have been attributed to the anti-inflammatory and antioxidative activities of scutellarin [ 18– 20] . Interestingly, our previous studies revealed that scutellarin not only inhibits canonical NLR family pyrin domain containing 3 (NLRP3) inflammasome activation but also suppresses noncanonical NLRP3 activation mediated by caspase-11, thus inhibiting pyroptosis [ 21, 22] . One recent study revealed that scutellarin inhibits pyroptosis via the selective degradation of p30/gasdermin D through autophagy [23]. Furthermore, we recently reported that scutellarin can inhibit PANoptosis in macrophages, thereby conferring protection against multi-organ injury in a mouse model of hemophagocytic lymphohistiocytosis that is associated with PANoptosis [24]. Given that ferroptosis is a prominent form of regulated cell death in AKI [ 16, 17] , it is of interest to investigate whether scutellarin can affect ferroptosis both in cultured cells *in vitro* and in mouse models of AKI *in vivo*.

In this study, we found that the flavonoid scutellarin effectively inhibited ferroptosis in human and mouse cells in response to the suppression of GPX4 or system X _c_^–^. This effect of scutellarin was mediated by increasing the antioxidant capacity of Nrf2. Scutellarin mitigated renal damage in a mouse model of AKI, which was associated with the suppression of ferroptotic signaling, likely through the upregulation of the Nrf2-GPX4 axis.

## Materials and Methods

### Reagents and antibodies

Scutellarin (B21478) was purchased from Shanghai Yuanye Bio-Technology Co. (Shanghai, China), dissolved in dimethyl sulfoxide (DMSO) at a concentration of 100 mM, and stored at ‒80°C. High-glucose Dulbecco’s modified Eagle’s medium (DMEM; C11995500BT), DMEM/F12 (C11330500BT), fetal bovine serum (FBS) (10099141C), penicillin-streptomycin (15140122), C11-BODIPY 581/591 (D3861), and MitoSOX Red (M36008) were obtained from Thermo Fisher Scientific (Carlsbad, USA). Folic acid (F7876), DMSO (D8418), propidium iodide (PI; P4170), Hoechst 33342 (B2261), Tween-80 (P8074), Tween-20 (P1379), DL-dithiothreitol (DTT; D0632), and CF488-conjugated goat-anti-mouse IgG (SAB4600237) were purchased from Sigma-Aldrich (St Louis, USA). Antibodies against Nrf2 (#12721), lamin A/C (#4777), β-tubulin (2128), β-actin (#3700) and HO-1 (#43966) were obtained from Cell Signaling Technology (Danvers, USA). The antibody against GPX4 (ab125066) was purchased from Abcam (Cambridge, UK). An anti-4-hydroxynonenal (4-HNE) antibody (JAI-MHN-020P) was purchased from AdipoGen (Liestal, Switzerland). An AlexaFluor647-conjugated anti-mouse/human MAC-2 antibody was obtained from BioLegend (San Diego, USA). Erastin (S7242), brusatol (S7956) and RSL3 (S8155) were purchased from Selleck (Houston, USA). Ferrostatin-1 (HY-100579) was obtained from MedChemExpress (Monmouth Junction, USA). WST-1 reagent (11644807001) was purchased from Roche (Mannheim, Germany). A nuclear and cytoplasmic protein extraction kit (P0027) was obtained from Beyotime (Shanghai, China). A creatinine assay kit (C011-2-1) was purchased from Nanjing Jiancheng Bioengineering (Nanjing, China). A blood urea nitrogen (BUN) kit (E2020) was obtained from Applygen (Beijing, China).

### Animals

C57BL/6J mice (6–8 weeks of age) were obtained from Guangzhou Ruige Biological Technology (Guangzhou, China). All the mice were housed under controlled conditions at 24 ± 2°C with a 12/12 h light-dark cycle and were provided *ad libitum* access to food and water. The animal experimental procedures were approved by the Jinan University Laboratory Animal Welfare and Ethics Committee and conducted in accordance with the committee’s guidelines.

### Cell culture and treatments

The human renal proximal tubular epithelial cell line HK-2 was a gift from Dr Liang Yan (the First Affiliated Hospital of Jinan University, Guangzhou, China). The cells were maintained in DMEM/F12 supplemented with 10% FBS and 1% penicillin-streptomycin (complete DMEM/F12) and subcultured at a 1:3 or 1:4 ratio every 2‒3 days. For the experiments, HK-2 cells were seeded at 1.5 × 10 ^5^ cells/well (0.5 mL) in 24-well plates or 6×10 ^5^ cells/well (1.7 mL) in 6-well plates in complete DMEM/F12 medium and incubated overnight at 37°C.

Bone marrow-derived macrophages (BMDMs) were isolated and cultured as previously described [25]. Briefly, bone marrow cells were flushed from femurs and tibias with 20 mL of sterile PBS, centrifuged at 300 *g* for 5 min at 4°C, and resuspended in BM-Mac medium (80% complete DMEM + 20% M-CSF-conditioned medium from L929 cells). The cells were cultured in 10-cm dishes with 10 mL of BM-Mac medium for 6 days in a humidified 37 °C incubator with 5% CO _2_ supplemented with 5 mL of fresh BM-Mac medium on day 3. For the experiments, BMDMs were seeded at 2.5 × 10 ^5^ cells/well (0.5 mL) in 24-well plates or 1.6 × 10 ^6^ cells/well (1.7 mL) in 6-well plates using complete DMEM and incubated overnight at 37°C.

### Cytotoxicity assay

The cytotoxicity of scutellarin was evaluated by using a WST-1 assay. In brief, BMDMs (2.5 × 10 ^5^ cells/well) or HK-2 cells (3 × 10 ^4^ cells/well) were seeded in 96-well plates (100 μL/well) and cultured at 37°C under 5% CO _2_ overnight. The cells were then treated with different concentrations of scutellarin for 24 h. Thereafter, 10 μL of WST-1 was added to each well and incubated at 37°C for 2 h. The absorbance at 450 nm was measured, and cell viability was evaluated and is presented as a percentage of the control.

### Cell death assay

Cell death was determined as described previously [26]. BMDMs or HK-2 cells were seeded in 24-well plates and incubated overnight. After the indicated treatments, the cells were incubated with freshly prepared staining solution containing PI (2 μg/mL) and Hoechst 33342 (5 μg/mL) for 10 min (0.5 mL/well; 37°C, 5% CO _2_). Fluorescence imaging was performed using an inverted fluorescence microscope (Axio Observer D1; Carl Zeiss, Göttingen, Germany) with Rhodamine (PI signal) and DAPI (Hoechst signal) channels. Images of 5 random fields were captured for statistical analysis. The percentages of cell death were determined by calculating the ratio of PI-positive nuclei (indicating dying cells) to Hoechst 33342-positive nuclei (total cells).

### Western blot analysis

Western blot analysis was performed as described previously [24]. Proteins from whole-cell lysates or nuclear proteins isolated by using the nuclear protein extraction kit (P0027; Beyotime) were separated by SDS-PAGE and transferred onto PVDF membranes (03010040001; Roche). The membranes were blocked with blocking buffer for 1 h at room temperature, followed by incubation with the following primary antibodies: Nrf2 (1:1000), HO-1 (1:2000), and GPX4 (1:8000). β-Actin (1:2000) was detected as a loading control. After overnight incubation at 4°C, the membranes were washed with PBS containing 0.05% Tween-20 (PBST) and incubated with an HRP-conjugated secondary antibody for 1 h at room temperature. The protein bands were visualized using an enhanced chemiluminescence kit (BeyoECL Plus, P0018; Beyotime, Shanghai, China) and X-ray film (RX-N; Fujifilm, Tokyo, Japan). Imaging and densitometric analysis were performed using a FluorChem 8000 system (AlphaInnotech, San Leandro, USA) with ImageJ and GraphPad Prism 7.0 software (GraphPad Software Inc., San Diego, USA).

### Measurement of mitochondrial superoxide

Mitochondrial superoxide levels (reflected by mitochondrial ROS, mtROS) were assessed by staining live cells with 3 μM MitoSOX Red. After 15 min of incubation, the fluorescence signals were visualized via an Axio Observer D1 fluorescence microscope.

### Assessment of the mitochondrial membrane potential

The mitochondrial membrane potential (MMP) was evaluated by using 5 μg/mL JC-1 (C2006; Beyotime) or a TMRE kit (C2001S; Beyotime) according to the protocols provided by the supplier. The cells were incubated with the fluorophores for 30 min, followed by live fluorescence imaging using an Axio Observer D1 microscope.

### Lipid peroxidation product determination

Lipid peroxidation, a hallmark of ferroptosis, was assessed by staining with 2 μg/mL of C11-BODIPY 581/591. The cells were incubated with the fluorescent probe for 30 min, followed by live fluorescence imaging using an Axio Observer D1 microscope.

### Animal model of AKI

AKI was induced in female C57BL/6J mice through folic acid (FA) administration. The mice were randomly divided into four groups ( *n *= 5 per group): vehicle control, scutellarin alone, FA alone, and the scutellarin + FA combination. Scutellarin was prepared in PBS containing 2% Tween-80 as a vehicle. The scutellarin and scutellarin + FA groups received an oral gavage of scutellarin solution (200 mg/kg body weight) once a day for two consecutive days. On day 3, these groups received an additional scutellarin dose 3 h prior to intraperitoneal FA injection (250 mg/kg body weight). The FA and scutellarin + FA groups were then administered with a FA solution dissolved in 0.3 M NaHCO _3_ solution. Forty-four hours after FA injection, the mice were anaesthetized with ethyl ether for orbital blood collection. Blood samples were used to prepare serum by centrifugation (300 *g*, 4°C for 30 min) after incubation at room temperature for 60 min followed by overnight incubation at 4°C. After euthanasia via cervical dislocation, kidney tissues were harvested for subsequent analyses.

### Serum creatinine and BUN measurement

Serum creatinine and BUN levels were determined using commercial assay kits according to the manufacturers’ protocols. In brief, serum was prepared from blood sample by centrifugation at 300 *g*, 4°C for 30 min. To detect creatinine and BUN levels, 6 μL serum per well and 3 μL serum per well were added to 96-well plates, respectively. Detection reagents were then added following the manufacturer’s instructions. The absorbance at 540 nm (for creatinine) or 490 nm (for BUN) was measured by using a microplate reader (Multiskan FC; Thermo Fisher Scientific). Creatinine and BUN concentrations were calculated based on their standard curves, respectively.

### Immunofluorescence staining

The left kidneys were fixed in 4% paraformaldehyde for 24 h (fully submerged) and frozen sectioned. The tissue sections were stored at ‒80°C until use. For antigen retrieval, the sections were equilibrated in PBS (10 min, room temperature) followed by immersion in preheated sodium citrate buffer (10 mM, pH 6.0, 80°C, 30 min). After cooling to room temperature, the sections were washed three times with PBS (5 min each) and circumscribed with an immunohistochemical pen. Blocking was performed via the addition of PBS containing 5% goat serum and 0.3% Triton X-100 (60 min, room temperature). Primary antibodies were applied within demarcated areas, and the samples were incubated at 4°C overnight. Following PBS washes, the corresponding fluorescent secondary antibodies were added, and the samples were incubated for 1 h at room temperature. After Hoechst 33342 nuclear staining (5 μg/mL PBS, 15 min), the slides were mounted with anti-fade medium. Images were captured via a Zeiss AxioCam MR R3 CCD camera controlled by ZEN software (Carl Zeiss).

### Histochemical analysis

The right kidneys were fixed in 10% neutral formalin for 24 h (fully submerged), and paraffin sections were routinely prepared. Images of the tissue sections were captured via an Axio Observer D1 microscope.

### Statistical analysis

The experiments were performed three times independently. Data are presented as the mean ± standard deviation (SD). Statistical significance was analyzed using GraphPad Prism 7.0. One-way ANOVA with the Bonferroni *post hoc* correction and unpaired Student’s *t* test were used for multi-group and two-group comparisons, respectively. Significance thresholds were defined as *P < *0.05.

## Results

### Scutellarin inhibits ferroptosis in both mouse and human cells

**Figure 1:**
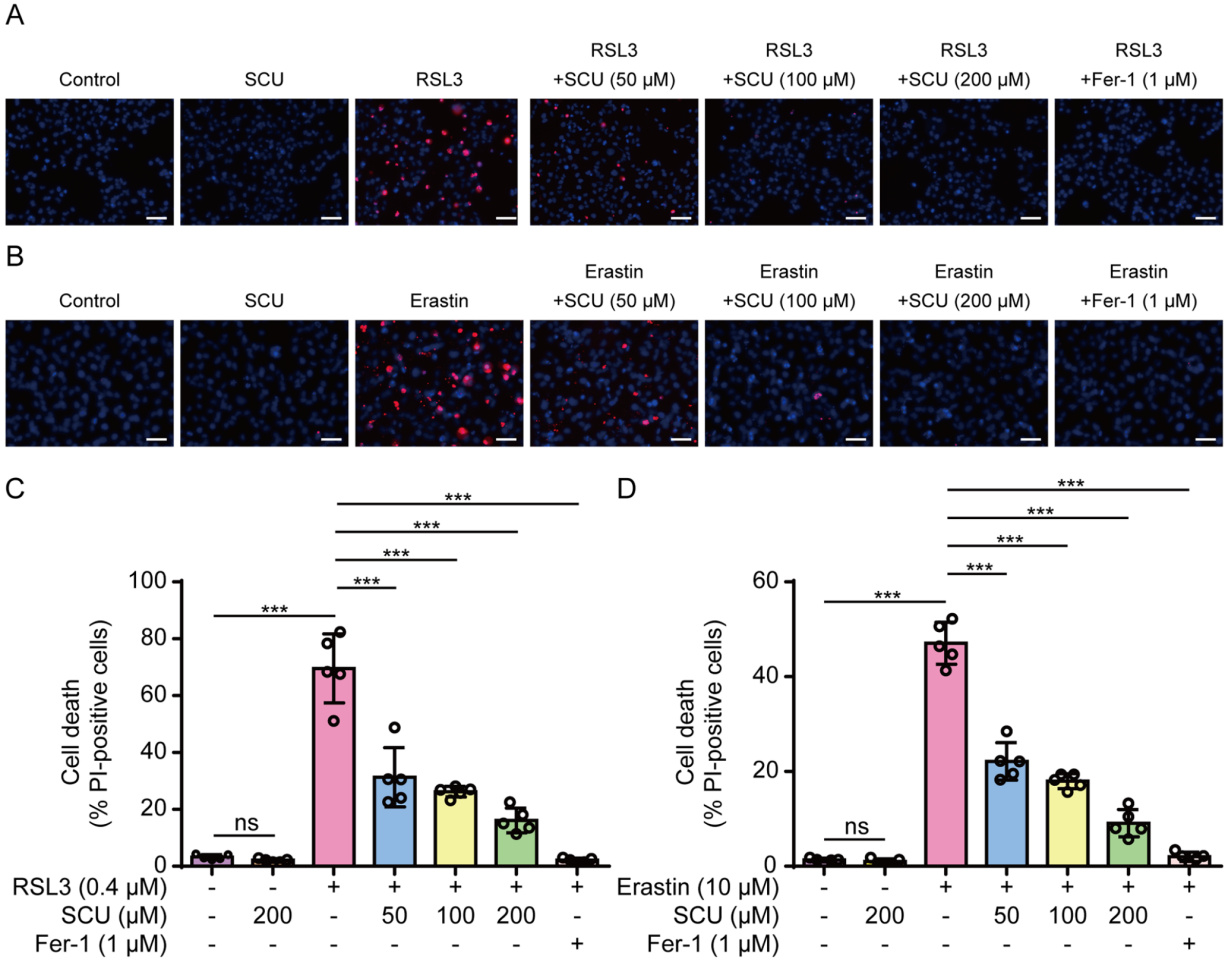
Figure 1 RSL3- or erastin-induced ferroptosis in HK-2 cells is inhibited by scutellarin HK-2 cells were pretreated with or without graded concentrations of scutellarin (SCU) for 1 h, followed by treatment with RSL3 (0.4 μM) for 5 h (A,C) or with erastin (10 μM) for 5 h (B,D). Cell death was assessed by propidium iodide (PI) staining (red, indicating dying cells) and Hoechst 33342 (blue, staining all nuclei). (A,B) Representative images were captured using fluorescence microscopy. Scale bar, 50 μm. (C,D) Quantitative analysis of the percentages of cell death. PI-positive cells were quantified in five random fields, and the percentage of cell death is presented as the ratio of PI-positive cells to total cells (revealed by Hoechst 33342 staining). Data are presented as the mean ± standard deviation (SD) (n = 5). ***P < 0.001; ns, not significant.

**Figure 2:**
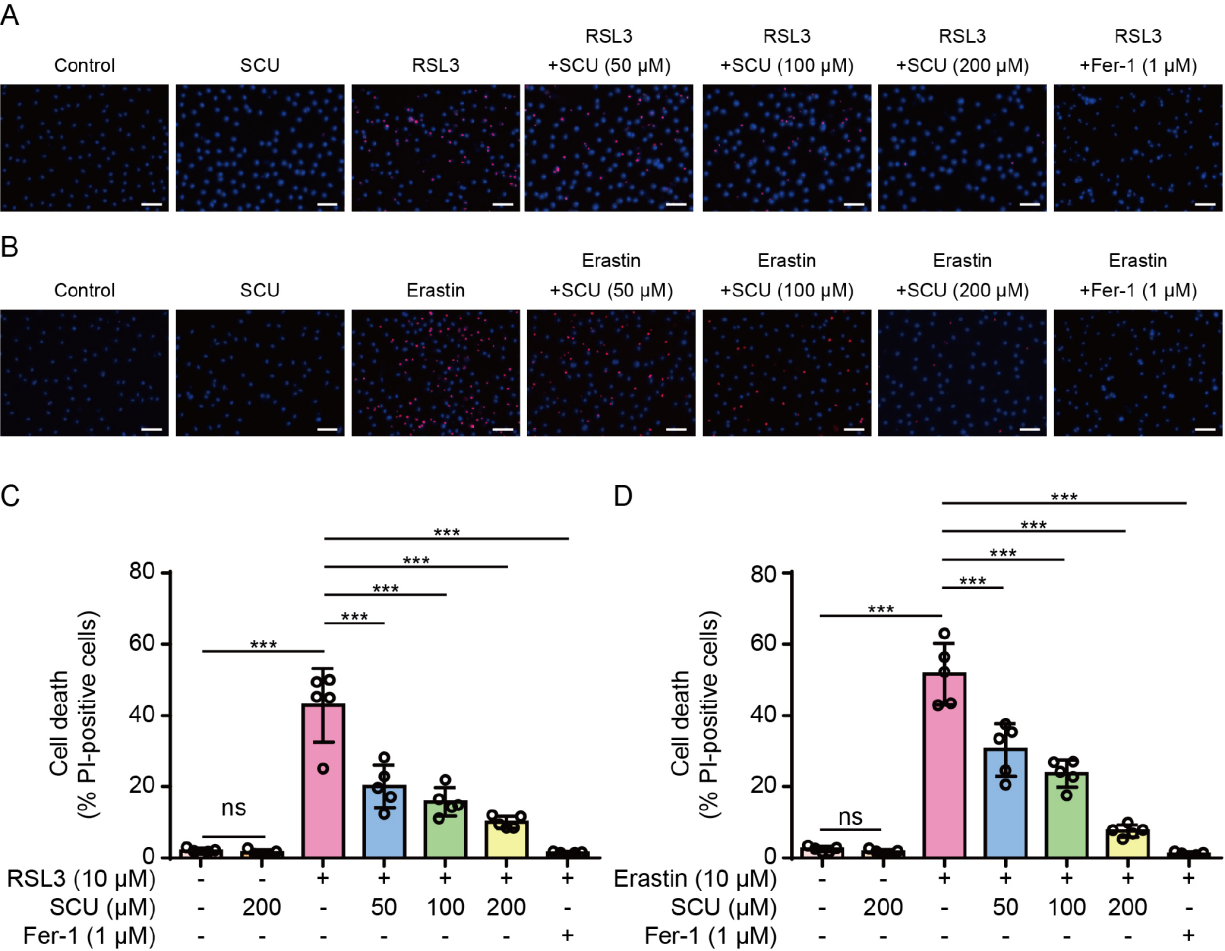
Figure 2 Ferroptosis in macrophages induced by RSL3 or erastin is inhibited by scutellarin Bone marrow-derived macrophages (BMDMs) were pretreated with or without graded concentrations of SCU for 1 h, followed by treatment with RSL3 (10 μM) for 24 h (A,C) or with erastin (10 μM) for 24 h (B,D). Cell death was assessed by PI staining (red, indicating dying cells) and Hoechst 33342 (blue, staining all nuclei). (A,B) Representative images were captured using fluorescence microscopy. Scale bar, 50 μm. (C,D) Quantitative analysis of the percentages of cell death. PI-positive cells were quantified in five random fields, and the percentage of cell death is presented as the ratio of PI-positive cells to total cells (revealed by Hoechst 33342 staining). Data are presented as the mean ± SD (n = 5). ***P < 0.001; ns, not significant.

As the antioxidant GPX4 and X _c_^–^ systems play essential roles in regulating ferroptosis, the pharmacological inhibition of GPX4 and X _c_^–^ by RSL3 and erastin, respectively, can induce this form of cell death [ 1, 5] . Thus, we assessed the effects of scutellarin on ferroptosis in the human renal proximal tubule epithelial cell line HK-2 and in mouse BMDMs treated with RSL3 or erastin. PI staining was used to detect cell death in these cells. RSL3 or erastin treatment markedly increased the percentage of PI-positive cells (indicative of dying cells), whereas scutellarin co-treatment significantly decreased the percentage of PI-positive cells ( Figure 1A–D). RSL3- or erastin-induced cell death was blocked by co-treatment with ferrostatin 1 (Fer-1), confirming ferroptotic cell death. Similar results were obtained from BMDMs ( Figure 2A–D). Scutellarin (200 μM) alone did not induce cell death in either HK-2 cells or BMDMs, which was verified by a cytotoxicity assay, which revealed that scutellarin was nontoxic at 200 μM or less ( Supplementary Figure S1A,B). Taken together, these results indicate that scutellarin inhibits ferroptosis in both human and mouse cells.

**Figure 3:**
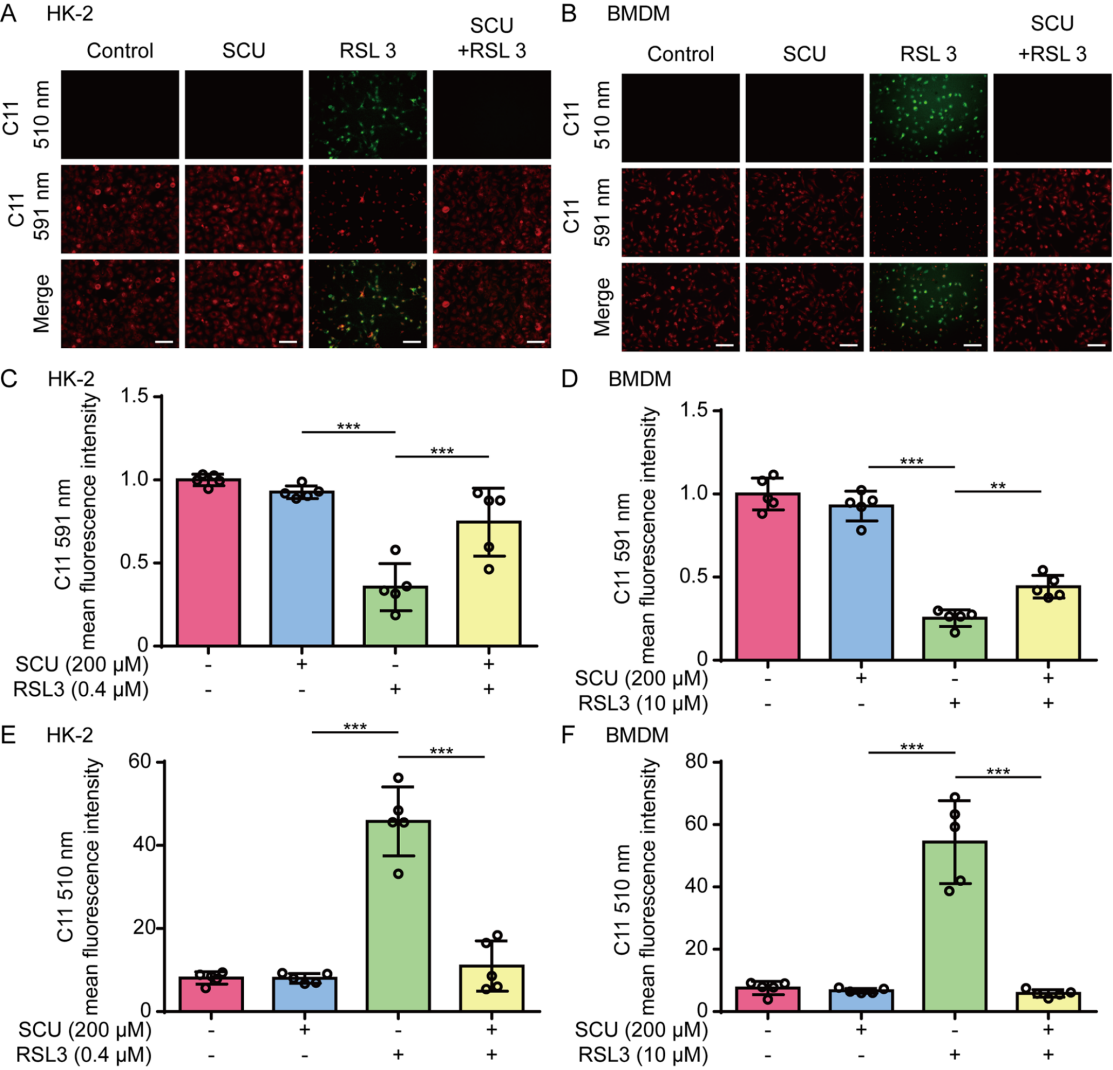
Figure 3 Lipid peroxidation in HK-2 cells and macrophages induced by RSL3 is inhibited by scutellarin HK-2 cells or BMDMs were pretreated with or without SCU (200 μM) for 1 h, followed by treatment with RSL3 (0.4 μM) for 5 h (A,C,E) or with RSL3 (10 μM) for 24 h (B,D,F), respectively. Lipid peroxidation was assessed via C11-BODIPY581/591 (5 μM) staining. The cells labelled with C11-BODIPY581/591 were excited at 488 nm and 561 nm to observe green and red fluorescence, respectively. (A,B) Representative images were captured via fluorescence microscopy. Scale bar, 50 μm. (C,D) Quantitative analysis of the red fluorescence intensity of (A,B). (E,F) Quantitative analysis of the green fluorescence intensity of (A,B). Data are presented as the mean ± SD (n = 5). **P < 0.01; ***P < 0.001.

We next assessed the effect of scutellarin on lipid peroxidation, which is a hallmark of ferroptosis [ 2, 3] . C11-BODIPY 581/591 dye is a sensor for lipid peroxidation [27]: C11 can be excited at 581 nm to emit red fluorescence (591 nm) in its reduced form; upon oxidation, the emission of red fluorescence is diminished, whereas the green fluorescence (510 nm) excited at 488 nm is increased [28]. Fluorescence microscopy revealed that RSL3 markedly increased green fluorescence and decreased red fluorescence in HK-2 cells and that these changes were reversed by co-treatment with scutellarin ( Figure 3A,C,E). Similar results were observed in BMDMs ( Figure 3B,D,F). Together, these results indicate that scutellarin can inhibit lipid peroxidation and ferroptosis in human and mouse cells.

### Scutellarin decreases mitochondrial damage during the induction of ferroptosis

**Figure 4:**
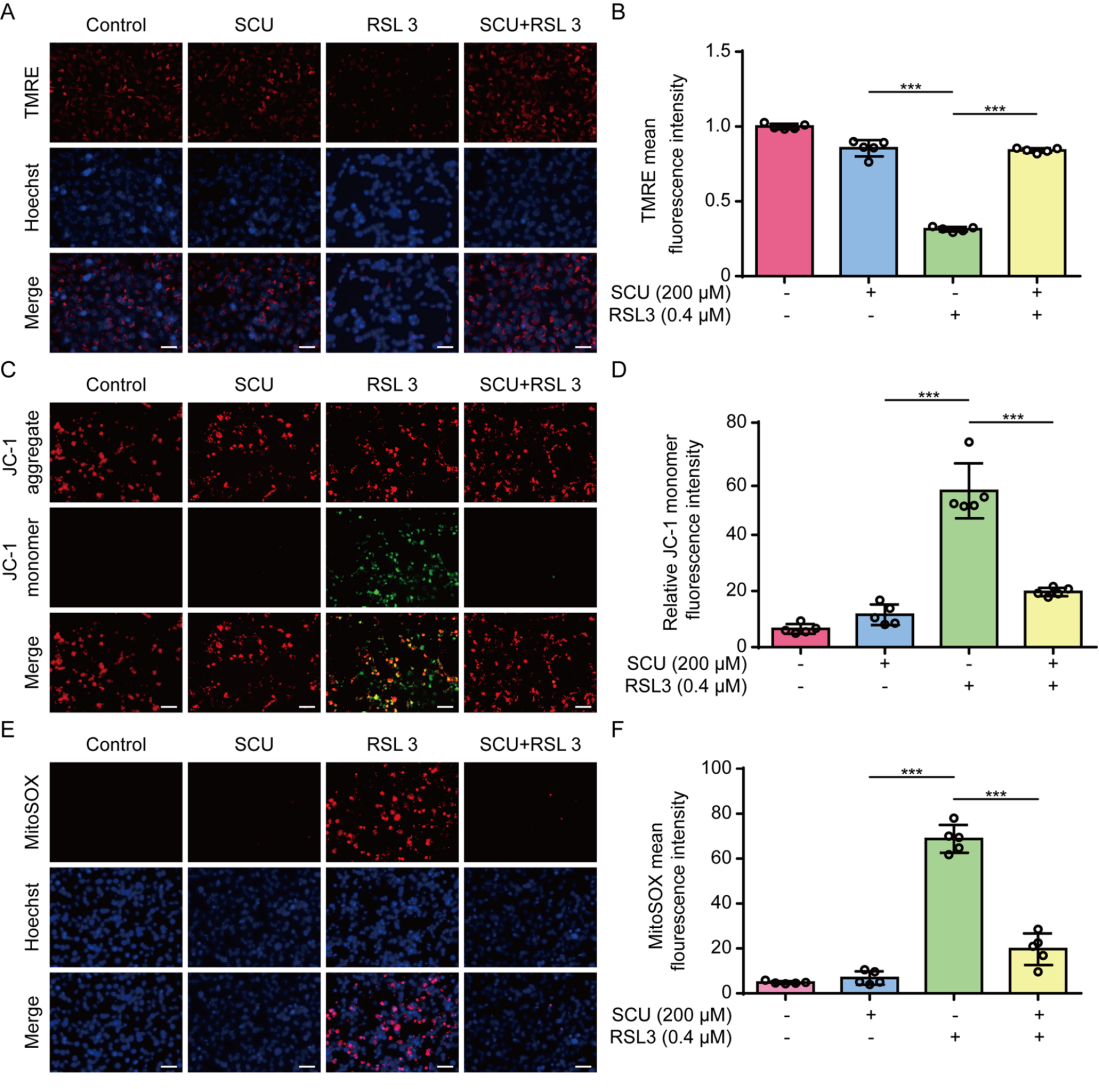
Figure 4 The RSL3-induced mitochondrial dysfunction in HK-2 cells is inhibited by scutellarin HK-2 cells were pretreated with different concentrations of SCU for 1 h, followed by treatment with RSL3 (0.4 μM) for 5 h. The mitochondrial membrane potential (MMP) was analyzed via TMRE (A,B) or JC-1 (C,D) staining. (A,C) Representative fluorescence images. (B,D) Quantitative analysis of TMRE and JC-1 in (A,C), respectively. (E,F) Mitochondrial superoxide (mtROS) levels were measured via MitoSOX staining, and images were captured using fluorescence microscopy (E). (F) Quantitative analysis of MitoSOX fluorescence. Data are presented as the mean ± SD (n = 5). Scale bar, 50 μm. ***P < 0.001.

**Figure 5:**
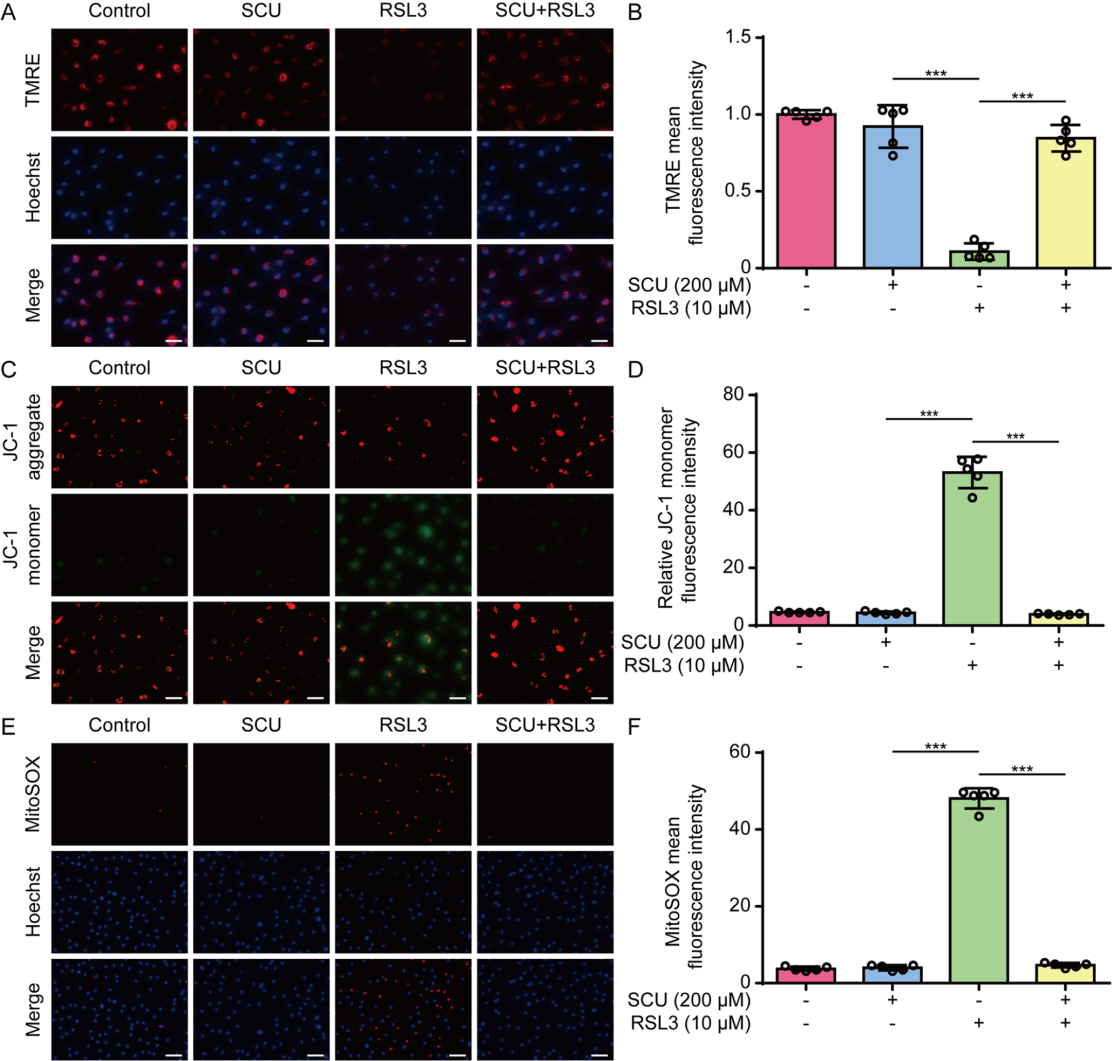
Figure 5 The RSL3-induced mitochondrial dysfunction in macrophages is suppressed by scutellarin BMDMs were pretreated with different concentrations of SCU for 1 h, followed by treatment with RSL3 (10 μM) for 24 h. The mitochondrial membrane potential (MMP) was analyzed via TMRE (A,B) or JC-1 (C,D) staining. (A,C) Representative fluorescence images. (B,D) Quantitative analysis of TMRE and JC-1 in (A,B), respectively. (E,F) Mitochondrial superoxide (mtROS) levels were measured by MitoSOX staining, and images were obtained using fluorescence microscopy (E). Quantitative analysis of MitoSOX fluorescence (F). Data are presented as the mean ± SD (n = 5). Scale bar, 50 μm. ***P < 0.001.

Given that mtROS play crucial roles in lipid peroxidation leading to ferroptosis [ 2, 9, 29– 31] , we next explored the effects of scutellarin on mtROS and mitochondrial function. Fluorescence microscopy revealed that TMRE fluorescence (indicative of the MMP) was decreased in HK-2 cells upon RSL3 treatment, whereas co-treatment with scutellarin reversed this decrease ( Figure 4A,B). JC-1 staining further confirmed a decrease in the MMP in HK-2 cells upon RSL3 stimulation, as revealed by decreased red fluorescence (indicating JC-1 aggregates within mitochondria) and increased green fluorescence (indicative of diffuse JC-1 monomers in the cytosol), which was counteracted by scutellarin ( Figure 4C,D). We also examined the production of mtROS by using MitoSOX staining and found that scutellarin significantly decreased MitoSOX fluorescence in HK-2 cells in response to RSL3 stimulation ( Figure 4E,F). Similar results were obtained in BMDMs ( Figure 5A–F). Taken together, these results indicate that scutellarin protects mitochondrial function and inhibits mtROS production during ferroptosis.

### Inhibition of ferroptosis by scutellarin relies on the upregulation of Nrf2 signaling

**Figure 6:**
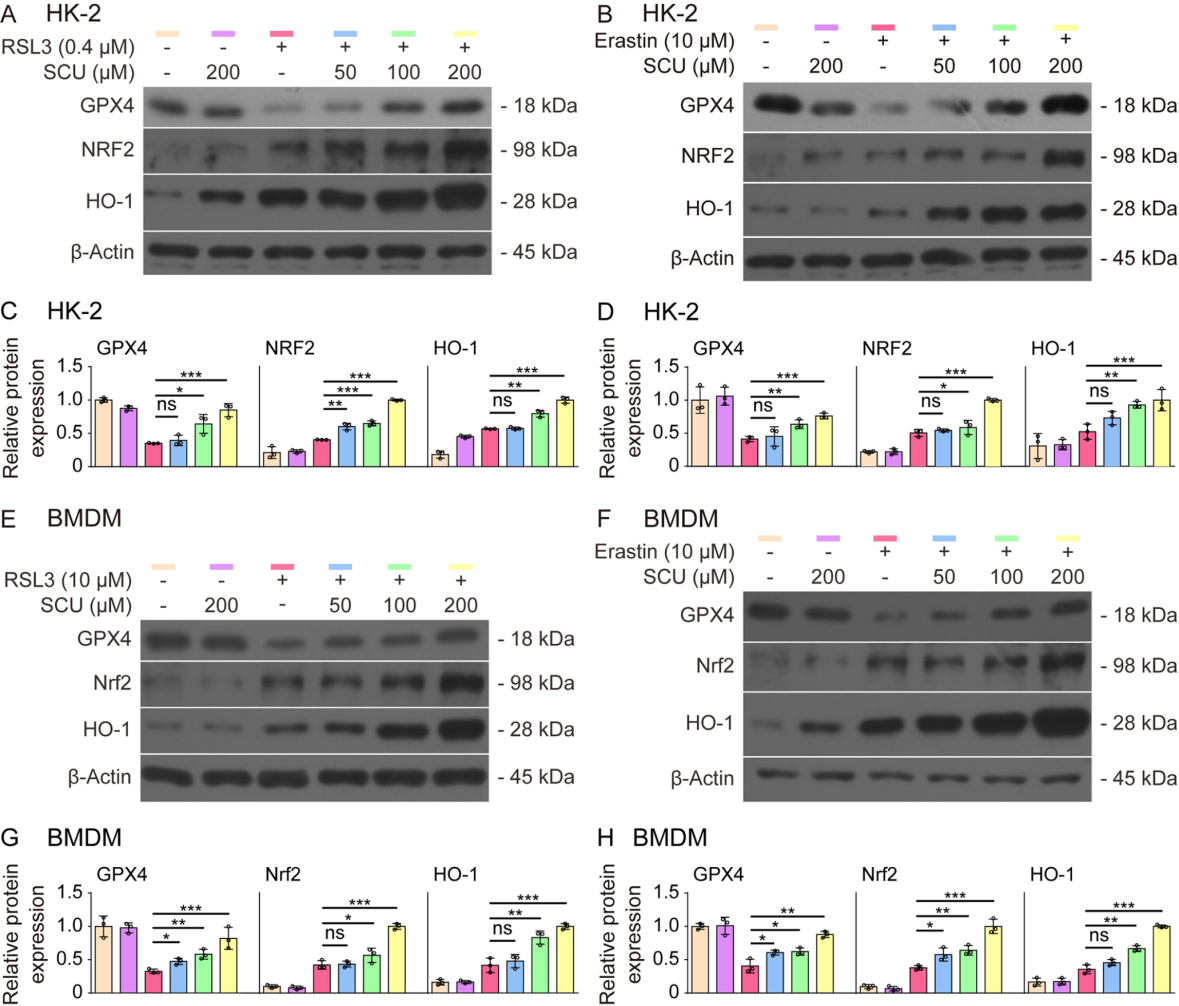
Figure 6 Scutellarin-mediated inhibition of ferroptosis is associated with the upregulation of Nrf2 signaling The cells were pretreated with different concentrations of SCU for 1 h, followed by treatment with RSL3 or erastin for 5 h (for HK-2 cells) or for 24 h (for BMDMs). (A,B,E,F) Western blot analysis of Nrf2, HO-1 and GPX4 levels in cell lysates. β-Actin was used as a loading control. (C,D,G,H) Quantitative analysis of protein expression in (A,B,E,F), respectively. Data are presented as the mean ± SD (n = 3). *P < 0.05; **P < 0.01; ***P < 0.001; ns, not significant.

As the Nrf2 transcription factor is known to control the expressions of many antioxidative proteins, including HO-1 and GPX4 [ 12, 13] , we next sought to investigate whether scutellarin could affect the expression of GPX4 through modulating Nrf2 activity. Accompanying the inhibition of ferroptotic cell death ( Figure 1), western blot analysis revealed that scutellarin counteracted the decrease in GPX4 induced by either RSL3 or erastin in HK-2 cells ( Figure 6A–D). Consistently, scutellarin markedly increased the levels of Nrf2 and HO-1 in HK-2 cells treated with RSL3 or erastin ( Figure 6A–D). Similar results were obtained with BMDMs ( Figure 6E–H). We further assessed whether scutellarin affects the nuclear translocation of Nrf2 by detecting its levels in isolated nuclear fractions. Western blot analysis revealed that scutellarin co-treatment with RSL3 or erastin dose-dependently increased Nrf2 levels in the nuclear fraction of HK-2 cells and BMDMs ( Supplementary Figure S2A–H). These results suggested that scutellarin increases the expression of GPX4 by upregulating Nrf2 signaling.

**Figure 7:**
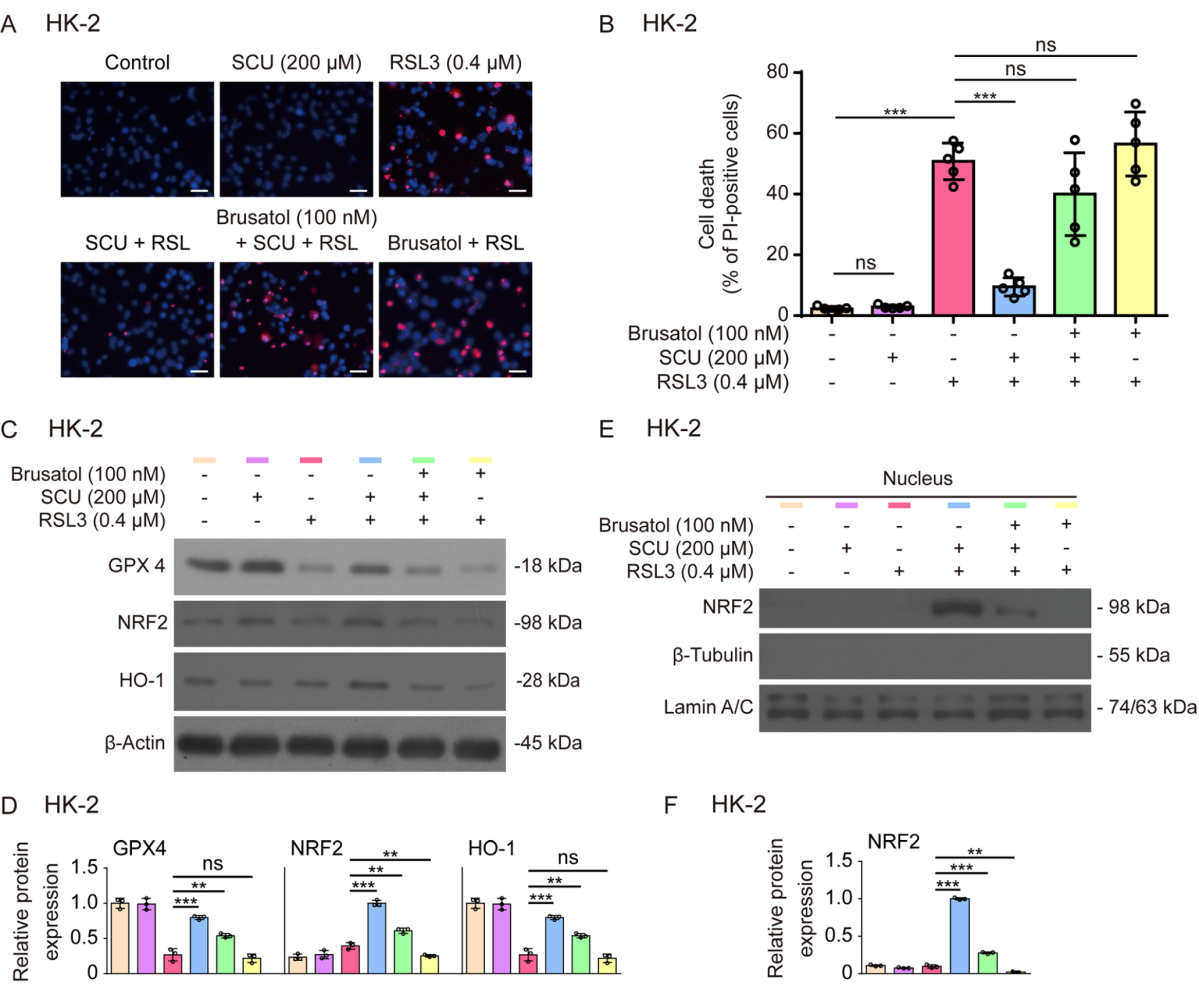
Figure 7 Scutellarin-mediated inhibition of ferroptosis is dependent on NRF2 signaling (A,B) HK-2 cells were pretreated with SCU alone or in combination with the NRF2-specific inhibitor brusatol for 1 h, followed by treatment with RSL3 for 5 h. Cell death was assessed by PI and Hoechst 33342 staining, and fluorescence images were captured using fluorescence microscopy (A). Scale bar, 50 μm. Quantitative analysis of percentages of cell death (B). Data are presented as the mean ± SD (n = 5). (C,D) Western blot analysis of NRF2, HO-1 and GPX4 levels in the presence or absence of brusatol (C) and quantitative analysis of the indicated proteins (D). (E,F) Immunoblot analysis of nuclear NRF2 protein levels in the presence or absence of brusatol (E) and quantitative analysis of NRF2 protein levels relative to lamin A/C (F). Lamin A/C was used as a loading control for the nuclear fraction. β-Tubulin was undetectable in the isolated nuclear fraction. Data are presented as the mean ± SD (n = 3). **P < 0.01; ***P < 0.001; ns, not significant.

To further verify the role of Nrf2 in mediating the inhibitory effect of scutellarin on ferroptosis, we co-treated HK-2 cells with scutellarin and brusatol, an Nrf2 inhibitor that enhances Nrf2 degradation [32], during ferroptosis. PI staining revealed that brusatol did not increase RSL3-induced cell death but significantly reversed the scutellarin-mediated inhibition of RSL3-induced ferroptotic cell death ( Figure 7A,B). Consistent with these findings, the scutellarin-mediated increases in NRF2, GPX4 and HO-1 in RSL3-treated cells were counteracted by brusatol co-treatment ( Figure 7C,D). We further assessed NRF2 levels in the nuclear fraction and found that scutellarin-induced NRF2 nuclear translocation was markedly suppressed by brusatol ( Figure 7E,F). These results indicate that scutellarin inhibits ferroptosis in HK-2 cells by upregulating Nrf2 signaling.

### Scutellarin administration mitigates folic acid-induced acute kidney injury

**Figure 8:**
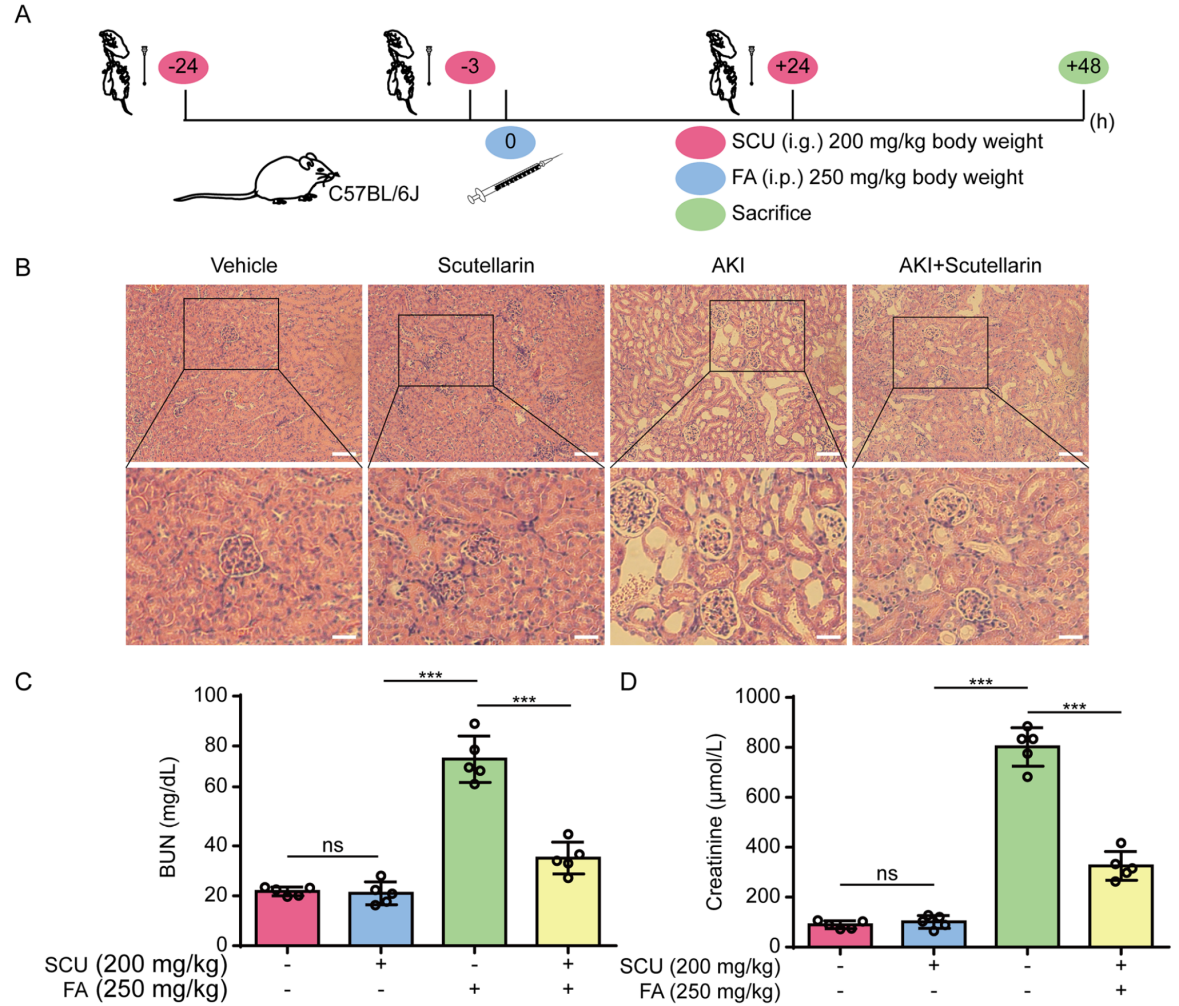
Figure 8 Folic acid-induced acute kidney injury is ameliorated by oral scutellarin administration (A) Schematic diagram depicting the SCU administration timeline for the folic acid (FA)-induced mouse model of AKI. (B) Histochemical analysis (H&E staining) of kidney tissue sections from the AKI model. Scale bars, 50 μm; 20 μm for insets. (C,D) Analysis of creatinine and blood urea nitrogen (BUN) levels. Data are presented as the mean ± SD (n = 5). ***P < 0.001; ns, not significant; i.p., intraperitoneal; i.g., intragastrical.

As ferroptosis is critical for renal injury in a mouse model of FA-induced AKI [33], we next assessed the effects of scutellarin on ferroptosis and renal injury in this mouse model. Acute renal injury was induced by a single dose of nephrotoxic FA intraperitoneally, and scutellarin was orally administered three times, as indicated in Figure 8A. Histochemical analysis of kidney sections revealed that FA induced marked pathological damage to the kidney, as revealed by pronounced vacuolization and increased Bowman’s space, which was ameliorated by scutellarin administration ( Figure 8B). This finding was further supported by the results of serum biochemical analyses, which revealed that scutellarin treatment significantly decreased the serum levels of BUN and creatinine in mice challenged with FA ( Figure 8C,D). Together, these results indicate that scutellarin can protect mice from FA-induced AKI.

### Mitigation of acute kidney injury by scutellarin is associated with decreased ferroptosis signaling

**Figure 9:**
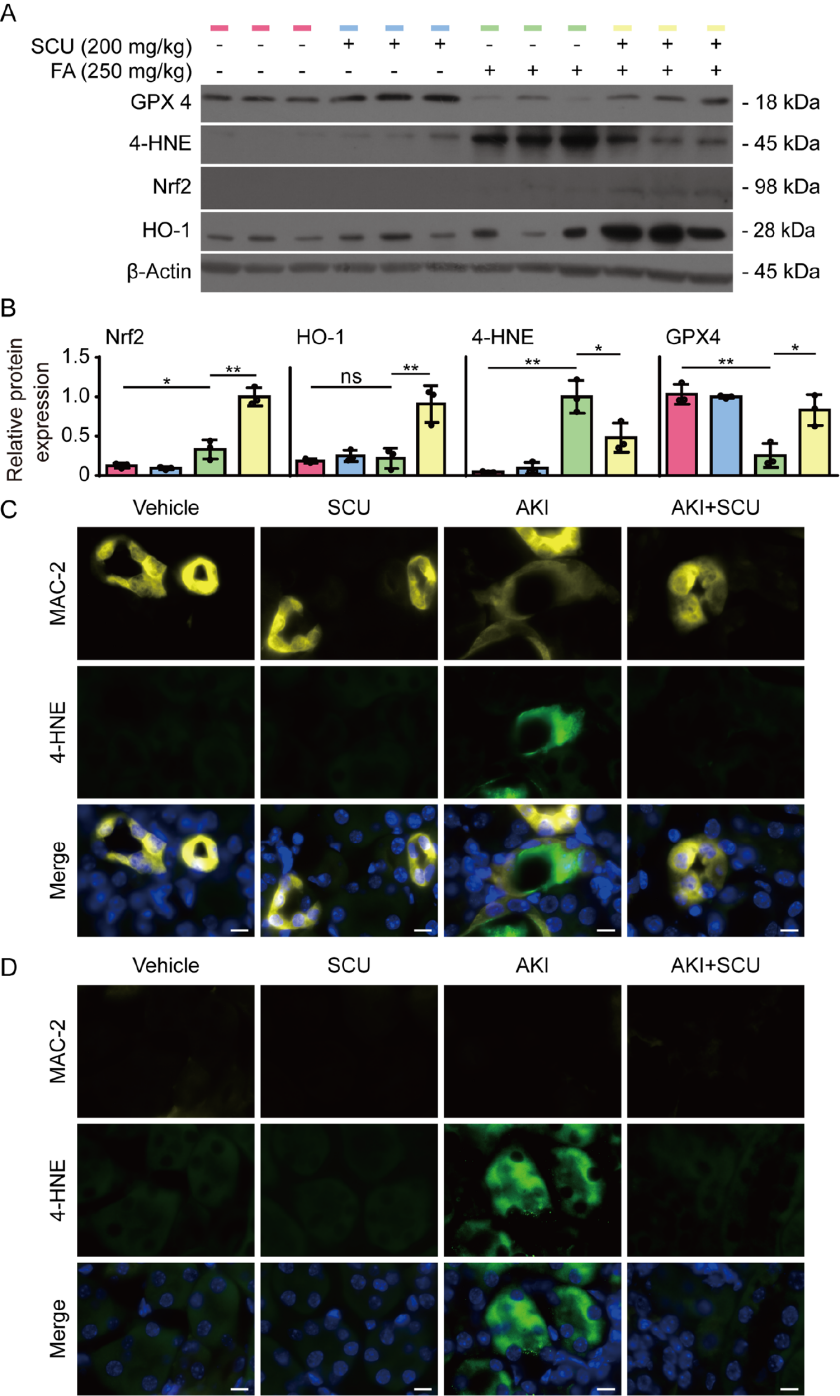
Figure 9 Mitigation of folic acid-induced acute kidney injury is associated with decreased levels of ferroptotic hallmarks in the kidney C57BL/6J mice were treated as shown in Figure 8A. (A) Western blot analysis was used to analyze the expressions of GPX4, 4-HNE, Nrf2, and HO-1 in the kidneys of 3 mice per group. β-Actin was used as a loading control. (B) Quantitative analysis of the relative expression levels of the proteins in (A). Data are presented as the mean ± SD (n = 3). *P < 0.05; **P < 0.01; ns, not significant. (C,D) Fluorescence microscopy revealed 4-HNE fluorescence in macrophages (MAC-2 positive) and other cells (MAC-2 negative) in the kidney. Kidney tissues were fixed with 4% paraformaldehyde, and frozen sections were prepared. After antigen retrieval, the tissue sections were stained with the indicated primary antibodies and corresponding fluorescent secondary antibodies. Immunofluorescence images were captured via fluorescence microscopy. Scale bar, 10 μm.

To further assess the associations between FA-induced AKI and ferroptosis, we analyzed the levels of GPX4, Nrf2, HO-1, and 4-HNE, the latter of which is a product of lipid peroxidation and is commonly used as a surrogate marker of ferroptosis [ 11, 34] . Western blot analysis revealed that FA significantly decreased the levels of GPX4 in the kidney and markedly increased the levels of proteins modified by 4-HNE ( Figure 9A,B), indicating lipid peroxidation and ferroptotic cell death in the kidney. In contrast, scutellarin administration increased GPX4 but decreased 4-HNE levels, indicating diminished lipid peroxidation and ferroptosis. Furthermore, Nrf2 and HO-1 levels were increased by scutellarin in the kidneys of mice treated with FA ( Figure 9A,B). We further assessed ferroptosis at the cellular level by detecting 4-HNE via immunofluorescence staining. Fluorescence microscopy revealed that 4-HNE expression was markedly increased in renal macrophages (MAC-2-positive) and other cells ( Figure 9C,D), which was abrogated by scutellarin co-administration. Together, these results suggest that scutellarin mitigates FA-induced renal injury by inhibiting lipid peroxidation and ferroptosis in the kidneys of mice, which is likely mediated by the regulation of Nrf2 signaling.

## Discussion

Ferroptosis, a form of oxidative cell death that is caused by iron-dependent lipid peroxidation, can be induced by blocking cellular antioxidant systems such as the GSH/GPX4 axis, and has been implicated in various pathological conditions, including acute kidney injury [ 4, 6, 14] . In this study, we explored the effects of scutellarin on ferroptosis in human HK-2 cells and mouse BMDMs treated with RSL3 or erastin. Our results showed that the flavonoid scutellarin inhibited ferroptosis in these cells treated with either RSL3 or erastin *in vitro* and mitigated FA-induced renal injury in a mouse model of AKI. Our study highlights scutellarin as an inhibitor of ferroptosis and thus has potential applications for the treatment of ferroptosis-related renal injury.

Scutellarin is a flavonoid that is the major ingredient of breviscapine used for the treatment of cerebrovascular and cardiovascular diseases [19]. Previous studies have indicated that scutellarin may exert its therapeutic effects on these diseases through its anti-inflammatory and antioxidative activities [18]. The therapeutic effects of scutellarin may also be mediated by its ability to inhibit inflammasome-induced pyroptosis [ 21– 23] . Our current study thus highlights another layer of the therapeutic mechanism by which scutellarin inhibits oxidative cell death—ferroptosis. Scutellarin inhibited ferroptosis in HK-2 cells and macrophages treated with either the GPX4 inhibitor RSL3 or the system X _c_^–^ inhibitor erastin. Interestingly, scutellarin inhibited lipid peroxidation, as revealed by C11-BODIPY 581/591 staining, suggesting that scutellarin might act on upstream targets of ferroptotic pathways. Indeed, we found that scutellarin could counteract the RSL3- and erastin-induced decrease in GPX4, the central antioxidant enzyme that can terminate the propagation of lipid peroxides [3]. Furthermore, our data revealed that scutellarin increased GPX4 level by increasing Nrf2 signaling, as blocking the activity of this transcription factor with the Nrf2-specific inhibitor brusatol abrogated the effects of scutellarin. This finding is consistent with previous studies showing that scutellarin can upregulate Nrf2 activity [ 35, 36] . In support of our results, the gene encoding GPX4 has been shown to be the target gene regulated by Nrf2 [ 12, 13] . In addition, the genes encoding components of system X _c_^–^ are also regulated by Nrf2 [ 12, 13] , but it is not known whether scutellarin affects system X _c_^–^. Together, our data reveal that scutellarin can counteract ferroptosis by increasing Nrf2 activity and thereby increasing the antioxidant capacity of the cell.

Mitochondria play critical roles in regulating ferroptosis [ 9, 30, 37] . mtROS contribute to ferroptosis by promoting lipid peroxidation [29]. Consistent with these findings, we found that the MMP was significantly reduced concomitant with increased production of mtROS during the induction of ferroptosis, whereas scutellarin markedly suppressed mtROS production and prevented MMP reduction accompanied by the inhibition of ferroptosis. How does scutellarin protect against mitochondrial dysfunction? One possibility is that scutellarin upregulates the Nrf2 transcription factor, as mentioned above, thereby increasing the levels of antioxidants, such as GPX1 and HO-1, in the cell to protect the mitochondria. Notably, DHODH operates in the mitochondrial inner membrane in parallel with mitochondrial GPX4 to inhibit ferroptosis by reducing ubiquinone to ubiquinol in certain cancer cells [9]; however, whether scutellarin can regulate this mitochondrial axis warrants further investigation. The second possibility is that scutellarin may attenuate mitochondrial damage through diminishing mitochondrial glucose oxidation by targeting the pyruvate dehydrogenase kinase (PDK)-pyruvate dehydrogenase complex (PDC) axis, as reported previously [38]. A third possible mechanism is that scutellarin may act as a lipophilic radical-trapping antioxidant to terminate mitochondrial lipid peroxidation, which has been detected in mitochondria during ferroptosis [ 9, 10] . However, these previse actions of scutellarin need further clarification.

Our data show that scutellarin can inhibit ferroptosis by increasing the antioxidant capacity of the cell through activating Nrf2 signaling, which is different from commonly used ferroptosis inhibitors such as Fer-1 and liproxstatin-1. Fer-1 inhibits ferroptosis by acting as a powerful radical scavenger and chain-breaking antioxidant, thus preventing lipid peroxidation and ferroptotic cell death [39]. Another potent ferroptosis inhibitor, liproxstatin-1, inhibits lipid peroxidation and rescues GPX4 level, which can protect against lipid peroxidation [39]. Thus, scutellarin, a natural product, represents an alternative candidate with different mechanisms of action for modulating ferroptosis-related disorders.

Consistent with the *in vitro* results, our *in vivo* data revealed that scutellarin inhibited ferroptosis (as revealed by the expression of 4-HNE, a surrogate marker of ferroptosis) in macrophages and other renal cells in the kidneys of FA-treated mice. The inhibition of ferroptosis was associated with reduced renal injury and increased expressions of Nrf2, HO-1 and GPX4, suggesting that scutellarin might exert its antiferroptotic effects by promoting the antioxidant capacity of cells in the kidney. However, other mechanisms of scutellarin may also act *in vivo* in parallel with the upregulation of Nrf2 signaling.

Although our *in vivo* results showed that the oral administration of scutellarin was able to mitigate FA-induced AKI, likely through the inhibition of ferroptotic signaling, we currently lack direct evidence for the effective exposure of the kidney to scutellarin, which is a limitation of this study. Several previous studies have reported the pharmacokinetics and tissue distribution of scutellarin in mice, rats and humans following different routes of administration. For example, intravenously administered scutellarin (20 mg/kg body weight) can be distributed into various organs, including the kidney [40]. Another study showed that 10% of the drug might be secreted through urine after a single oral dose of scutellarin (400 mg/kg body weight) in rats, suggesting that a substantial part of the drug had been absorbed and eliminated by the kidney [41]. A clinical study further revealed that orally administered scutellarin (60 mg for an average body weight of 64 kg) can be absorbed into the blood even when the plasma concentration of scutellarin is low [42]. These studies suggest that scutellarin can be absorbed and redistributed into the kidney even though its bioavailability is low. Thus, multiple administrations of this drug seem necessary to achieve an effective concentration in targeted organs such as the kidney.

In conclusion, the present study reveals that the flavonoid scutellarin effectively inhibits ferroptosis *in vitro* in cultured cells and *in vivo* in the kidneys of mice with FA-induced AKI. The Nrf2 signaling pathway plays a critical role in mediating the antiferroptotic activity of scutellarin. Our data highlight that scutellarin can act as an inhibitor of ferroptosis to cope with ferroptosis-related diseases, including acute renal injury.

## Supporting information

25223Supplementary_figures

## Supplementary Data

Supplementary data is available at *Acta Biochimica et Biophysica Sinica* online.
