# Supplementary material for: Scutellarin inhibits ferroptosis by promoting cellular antioxidant capacity through regulating Nrf2 signaling: Scutellarin inhibits ferroptosis by promoting cellular antioxidant capacity
Source: Acta Biochim Biophys Sin (Shanghai). 2025 Aug 19;58(3):516–29. doi: 10.3724/abbs.2025112 (PMC13059782; doi:10.3724/abbs.2025112)
Supplement: 25223Supplementary_figures [file 25223Supplementary_figures.docx]

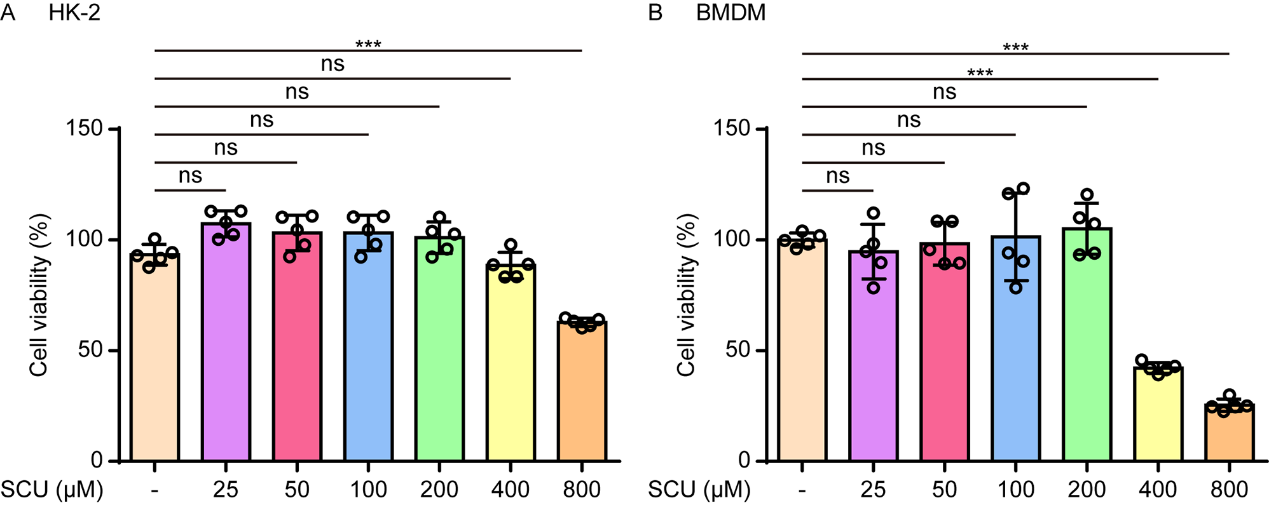


**Supplementary Figure S1. The cytotoxicity of scutellarin on HK-2 cells and bone marrow-derived macrophages (BMDMs)**  Cells were treated with different concentrations of scutellarin (SCU) for 24 h, followed by addition of WST-1 reagent. The absorbance at 450 nm was measured using a microplate reader. The cell viability was presented as percentages of control for HK-2 cells (A) and BMDMs (B). Data are presented as the mean ± SD (*n* = 5). ***P* < 0.01; ****P* < 0.001; ns, not significant.


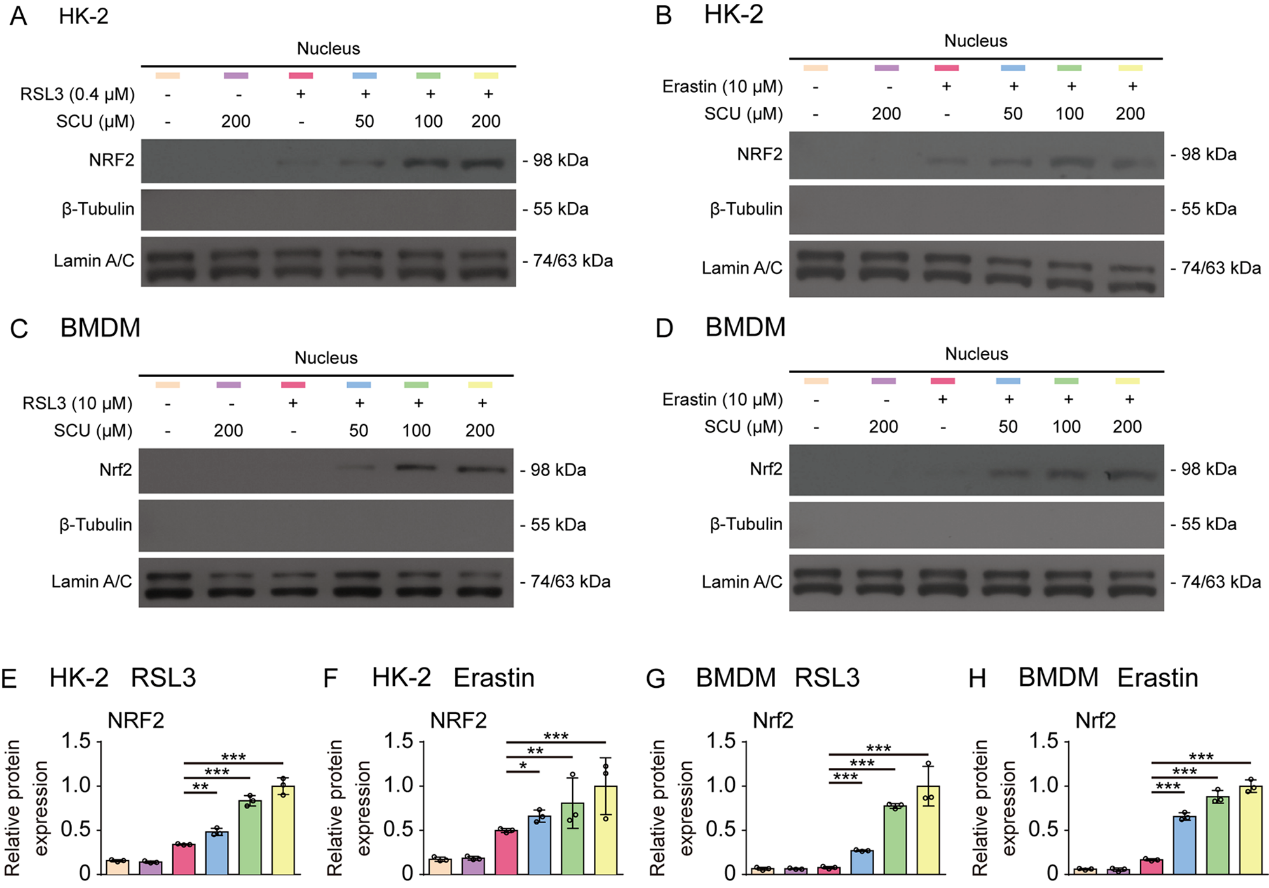


**Supplementary Figure S2.** **The effect of scutellarin on nuclear translocation of NRF2** Cells were pretreated with different concentrations of scutellarin (SCU) for 1 h, followed by treatment with RSL3 or erastin for 5 h (for HK-2) or 24 h (for BMDMs). (A−D) Western blot analysis of Nrf2 levels in the nuclear fraction. Lamin A/C were used as a loading control for the nuclear fraction. β-Tubulin was undetectable in the isolated nuclear fraction. (E−H) Quantitative analysis of protein levels of NRF2 relative to lamin A/C in (A−D), respectively. Data are presented as the mean ± SD (*n* = 3). **P* < 0.05; ***P* < 0.01; ****P* < 0.001.
